# Burst Release of Antibiotics Combined with Long-Term Release of Silver Targeting Implant-Associated Infections: Design, Characterization and *in vitro* Evaluation of Novel Implant Hybrid Surface

**DOI:** 10.3390/ma12233838

**Published:** 2019-11-21

**Authors:** Kai Borcherding, Dennis Marx, Linda Gätjen, Nicole Bormann, Britt Wildemann, Uwe Specht, Dirk Salz, Karsten Thiel, Ingo Grunwald

**Affiliations:** 1 Department of Adhesive Bonding Technology and Surfaces, Fraunhofer Institute for Manufacturing Technology and Advanced Materials (IFAM), 28359 Bremen, Germany; Dennis.Marx@ifam.fraunhofer.de (D.M.); Linda.Gaetjen@ifam.fraunhofer.de (L.G.); Uwe.Specht@ifam.fraunhofer.de (U.S.); Dirk.Salz@ifam.fraunhofer.de (D.S.); Karsten.Thiel@ifam.fraunhofer.de (K.T.); 2 Julius Wolff Institute, BIH Center for Regenerative Therapies, Charité—Universitätsmedizin Berlin, Corporate Member of Freie Universität Berlin, Humboldt-Universität zu Berlin, and Berlin Institute of Health, 13353 Berlin, Germany; Nicole.bormann@charite.de (N.B.); Britt.Wildemann@med.uni-jena.de (B.W.); 3 Experimental Trauma Surgery, Department of Trauma, Hand and Reconstructive Surgery, University Hospital Jena, 07747 Jena, Germany; 4 Industrial and Environmental Biology, Hochschule Bremen-City University of Applied Sciences, Neustadswall 30, 28199 Bremen, Germany; i.grunwald@hs-bremen.de

**Keywords:** implant-associated infection, silver, gentamicin, osseointegration, titanium, surface coating, orthopedics, release

## Abstract

Implant-associated infections represent a serious risk in human medicine and can lead to complications, revisions and in worst cases, amputations. To target these risks, the objective was to design a hybrid implant surface that allows a local burst release of antibiotics combined with long-term antimicrobial activity based on silver. The efficacy should be generated with simultaneous *in vitro* cytocompatibility. The investigations were performed on titanium K-wires and plates and gentamicin was selected as an illustrative antibiotic. A gentamicin depot (max 553 µg/cm^2^) was created on the surface using laser structuring. The antibiotic was released within 15 min in phosphate buffered saline (PBS) or agar medium. Metallic silver particles (4 µg/cm^2^) in a titanium dioxide layer were deposited using plasma vapor deposition (PVD). About 16% of the silver was released within 28 days in the agar medium. The local efficacy of the incorporated silver was demonstrated in a direct contact assay with a reduction of more than 99.99% (*Escherichia coli*). The local efficacy of the hybrid surface was confirmed in a zone of inhibition (ZOI) assay using *Staphylococcus cohnii.* The biocompatibility of the hybrid surface was proven using fibroblasts and osteoblasts as cell systems. The hybrid surface design seems to be promising as treatment of implant-associated infections, considering the achieved amount and release behavior of the active ingredients (gentamicin, silver). The generated *in vitro* results (efficacy, biocompatibility) proofed the concept. Further *in vivo* studies will be necessary translate the hybrid surface towards clinical applied research.

## 1. Introduction

Implant-associated infections [1] are still an unsolved problem in orthopedics, even when advanced materials and implants with local antibiotics are available [2]. Heitemayer and Hax [3] found that in the 1990s, the cost of the treatment for an implant-associated infection was five to seven and a half times higher than that of treatment without complications. For example, Arens [4] estimated the economic damage for such infections—only in the field of endoprosthetics—at approximately €150 million/year in Germany, and it is currently being assessed whether subsequent revisions lead to a financial loss in clinics [5,6]. In most cases, the pathogens are *Staphylococci* [7], especially *Staphylococcus aureus* [8], which cause orthopedic bone implant infections. Local antibiotics have significantly more potential to prevent infections than systemically applied antibiotics, as shown in animals [9] and humans [2]. Local antibiotics enable high concentrations at the infection site that are not possible with systemic antibiotics and also play a significant role against implant-associated infections [10]. Various approaches for the local delivery of antibiotics based on implant modifications are currently under investigation [11,12,13]. Meanwhile, established coatings for orthopedic applications primarily use gentamicin or silver. 

A hybrid implant surface has been developed that combines the strengths of two approaches, namely the local release of antibiotics and long-term antimicrobial action due to silver ions. Based on a broad experience with gentamicin in clinical applications, this approach was selected as an illustrative antibiotic. The target concentrations of the here described novel implant surface design were derived from the following rationale.

Gentamicin: The applicability of a local gentamicin coating was demonstrated for the Synthes product “Expert Tibia Nail PROtect” [14], which contains approx. 170 µg/cm^2^ gentamicin sulfate (102 µg/cm^2^ gentamicin base) in a polylactide coating [15]. The Synimed company applies an alternative approach for intramedullary nails using a coating of bone cement containing gentamicin (5000 µg/cm^2^ gentamicin sulfate), from which 31% is released within the first 30 days after implantation [16]. Based on this, an applicable gentamicin content is considered to be between 100 and 1500 µg/cm^2^ (gentamicin base). The target gentamicin concentration was selected in compliance with Palacos R+G bone cement experiments [17] and is thereby in the area of 400 µg/cm^2^ gentamicin base.

Silver: Silver coatings available on the market for orthopedics are, for example, “Agluna”, a silver ion depot with 4–6 µg/cm^2^ silver [18], “PorAg” a 1 µm thick silver layer combined with a top layer of TiAg20N (0.1 µm thick) [19], and a 10 to 15 µm thick silver coating combined with a 0.2 µm gold layer on a “MUTARS” megaprosthesis [20]. The efficacy of these selected coatings has been clinically demonstrated (“PorAg” [19], “AgLuna” [21] and “MUTARS” megaprosthesis [22]). In clinical applications, the remaining bacterial load on a gentamicin coated implant after implantation and release of the antibiotic is expected to be low. Consequently, the silver coating is considered a second line of defense against further bacterial colonization of the implant surface during osseointegration, and thus a comparable low target concentration was selected (silver: 4 µg/cm^2^). 

## 2. Materials and Methods 

This study aimed at the design, characterization and *in vitro* testing of a hybrid implant surface for local gentamicin and silver release.

### 2.1. Titanium Test Specimens

The following titanium grade 5 test specimens were used in this study: K-wire with a length of 150 mm and a diameter of 1.0 mm (mahe medical GmbH, Emmingen-Liptingen Germany) and square plate specimens (2 cm long, 2 cm wide and 1 mm high) (Rocholl GmbH, Aglasterhausen, Germany). In summary, the following K-Wire and plate groups were used:

- Unmodified K-wire or plate (referred to as “U K-wire or U plate”);
- K-wire or plate with a porous structure (referred to as “PS K-wire or PS plate”);
- K-wire or plate with a silver/titanium coating (referred to as “ST K-wire or ST plate”);
- K-wire or plate with a porous structure and a silver/titanium coating (referred to as “hybrid K-wire or hybrid plate”);
- K-wire with a porous structure, a silver/titanium coating and loaded with gentamicin (referred to as “G hybrid K-wire”).

### 2.2. Gentamicin Depot 

The laser treatment was carried out with a Q-switched Nd:YAG laser (l = 1064 nm) type CL100 with an average power of 100 W (Clean Lasersysteme GmbH, Herzogenrath, Germany). The laser operates in a frequency range between 100 and 200 kHz. Suitable carrier surfaces were achieved with a fluence of 5.3 J/cm² in the focal plane. The laser process created a porous structure on the surface, which increased the K-wire diameter (measured using an electronic measuring caliper). The loading procedure of the hybrid K-wire was performed with different gentamicin sulfate solution contents. Defined amounts of gentamicin sulfate (Carl Roth GmbH + Co. KG, Karlsruhe, Germany) were dissolved in phosphate buffered saline (PBS). The hybrid K-wires were placed in the solution for 5 min to infiltrate the structure. The adhering surface film was reduced to a constant minimum by pulling the G hybrid K-wire through polytetrafluoroethylene-faced butyl septum (Merck, Darmstadt, Germany). 

Gentamicin was quantified using ninhydrin [23]. Ninhydrin reacts with the primary and secondary amino groups of gentamicin to produce an optically measurable yellow and purple coloring agent. For a comparative quantification, the clinical routine quantification method known as “kinetic interaction of microparticles in solution” (KIMS) (Cobas Integra 400, Roche, Germany; Labor Berlin-Charité Vivantes GmbH, Berlin, Germany) was performed. The experiments with gentamicin sulfate in PBS revealed that the recovery rate in KIMS assay was 62.5% (validated method for human serum and in combination with neither PBS nor agar). All KIMS values were calculated accordingly. Two different concentrations were evaluated: nominal and high concentrations. Nominal concentration: 400 µg/cm^2^ was selected as the targeted gentamicin base concentration. The release over time and efficacy were evaluated for this concentration. The release was quantified in PBS and in the agar medium to mimic an artificial *in vivo* environment with a surrounding medium of higher density. Safe-Lock Tubes (Eppendorf, Hamburg, Germany) were filled with 0.5 mL 1.5% low melting agarose (Carl Roth GmbH + Co. KG, Karlsruhe Germany) prepared in PBS or were filled with PBS only. Gamma irradiated (31 kGy) hybrid K-wires were loaded with gentamicin sulfate (290 mg/mL), cut into 7 mm length and placed into the middle of the tubes. Samples were extracted after the time points 15 min, 30 min, 1 h, 4 h, 1 day, 3 days, and 7 days. The extracts with agar were heated to 85 °C for 30 min. Aliquots from the agar and PBS samples were used in ninhydrin and KIMS assays to determine the gentamicin base concentration. The extracted G hybrid K-wire pieces were subsequently tested in an agar diffusion test regarding their efficacy. A higher gentamicin concentration was tested, with the expectation that a higher concentration has an increased negative effect on cells. The sample preparation was performed as with the nominal concentration, except for the loading concentration (500 mg/mL gentamicin sulfate) and a constant release time of 30 min, which was used to evaluate the biocompatibility and influence of steam sterilization. 

### 2.3. Silver Depot

The silver depot was prepared on top of the lasered structure by embedding elemental silver particles in a titanium dioxide layer. The coating was implemented in a high frequency sputtering chamber fabricated in-house (Fraunhofer IFAM, Bremen, Germany). The titanium dioxide layer was deposited by reactive magnetron sputtering using a planar magnetron (VON ARDENNE GmbH, Dresden, Germany) and a 99.9% titanium target (Sindlhauser Materials GmbH, Kempten, Germany). The process was performed in a reduced atmosphere containing oxygen (99.998% purity) and argon (99.999% purity) (Linde AG, Pullach, Germany). The elemental silver particles were produced by sputtering a 99.99% silver target (Sindlhauser Materials GmbH, Kempten, Germany) in a pure argon atmosphere. The silver quantity and its release over time were quantified in an agar medium (1.5% low melting agarose in PBS, Carl Roth GmbH + Co. KG, Karlsruhe, Germany) to mimic an artificial *in vivo* environment. Gamma-irradiated hybrid K-wires were loaded with gentamicin sulfate (290 mg/mL) and placed into the center of the 3 mL vial. After the time points of 1 day, 7 days, and 28 days, the samples were extracted and dried at 120 °C for 15 min. For silver quantification, the samples were dissolved with diluted (33%) sub-boiled nitric acid and measured using an inductively coupled plasma atomic emission spectrometer (iCap 6500, Thermo Fisher Scientific, Bremen, Germany; Mikroanalytisches Labor Pascher, Remagen, Germany). 

### 2.4. Optical Characterization 

Optical micrographs of the surface topography were captured by a VHX-1000 digital microscope with a VH-Z 100 lens (Keyence Int. Trading Co. Ltd., Osaka, Japan). Nanostructural analyses of the silver depot were performed using transmission electron microscopy (TEM, Tecnai F20 S-TWIN microscope, FEI, Eindhoven, Netherlands). TEM samples were prepared via the focused ion beam (FIB) technique (Helios 600 dualbeam; FEI, Eindhoven, Netherlands). Elemental analysis was carried out by energy-dispersive X-ray spectroscopy (EDX) using the EDAX r-TEM-EDX-Detector of the TEM machine.

### 2.5. Sterilization Influence

The influence of gamma irradiation on the plasma vapor deposition (PVD) coating composition was assessed by TEM and EDX after applying a dosage of 31 kGy gamma irradiation (BGS Beta-Gamma-Service GmbH & Co. KG, Wiehl, Germany). All gamma-irradiated samples were packaged under vacuum (<5 mbar) in aluminum compound foils (Gruber-Folien GmbH & Co. KG, Straubingen, Germany). 

The influence of autoclaving (saturated steam at 121 °C for 20 min and drying for 30 min at 104 °C) on the gentamicin loading capacity was evaluated via ninhydrin assay.

### 2.6. Efficacy 

The antibacterial efficacy was evaluated against bacteria of risk group 1 (classification according to the German technical rules for biological agents). The majority of implant-associated infections are caused by *Staphylococci* (gram-positive) and *Escherichia coli* (gram-negative) [24]. According to the epidemiology and the limitation regarding risk classification, *Staphylococcus cohnii* (ATCC 29974) (*S. cohnii*) was selected for the efficacy study of the hybrid surface measuring zone of inhibition (ZOI). The G hybrid K-wire pieces used to assess gentamicin release over time were placed without any further preparation on 1.5% tryptic soy broth agar (Merck, Darmstadt; Germany), which was inoculated with 100 µL S. cohnii (2.25 × 10^7^ CFU/mL), and incubated for 24 h at 37 °C. The inhibition zone was measured using ImageJ software (Version 1.52a, Maryland, United States of America) and converted to ideal circle diameter. The antibacterial efficacy of the silver/titanium dioxide coating was evaluated according to ISO 22196. The square specimens were inoculated with 100 µL of an *Escherichia coli* (ATCC 15766) (*E. coli*) suspension (5 × 10^5^ CFU/mL), covered with sterile coverslips, and incubated for 24 h at room temperature. The coverslips were removed and 100 µL sterile PBS was mixed with the bacterial suspension. Subsequently, 100 µL of this suspension was transferred to the wells of a 96-well plate and 150 µL lysogeny broth medium was added. The bacterial growth was followed over a period of 48 h with a microplate reader (Mithras LB940, Berthold Technologies, Bad Wildbad, Germany) using optical density measurements at 620 nm. 

### 2.7. In vitro Cytotoxicity

The cytotoxicity was assayed according to the DIN EN ISO 10993-5 guidelines using the cleavage of the tetrazolium salt WST-1(4-(3-(4-Iodophenyl)-2-(4-nitrophenyl)-2H-5-tetrazolio)-1,3-benzene disulfonate) to formazan. Hybrid K-wires were loaded with a high concentration of gentamicin sulfate (500 mg/mL). The extraction was carried out in a 550 µL Roswell Park Memorial Institute (RPMI) 1640 medium at 37 °C, 24 h prior to the application of extracts on cells. L929 (mouse fibroblasts) and MG-63 (human osteoblast-like cells) (100 µL cell suspension, 10^5^ cells/mL) were seeded into a 96-well plate and incubated for 24 h at 37 °C, 5% CO_2_. An amount of 100 µL supernatant was substituted with 100 µL extract. After an additional 24 h of incubation, a WST-1 assay was conducted. Untreated cells were used as a negative control (cytocompatible), and a treatment with 10% hydroxyethyl methacrylate (HEMA) was used as a positive control (cytotoxic). Complementary to the WST-1 assay, optical micrographs were taken using an Axio Vert.A1 microscope (Carl Zeiss, Jena, Germany) for the direct morphological interpretation of cell viability.

### 2.8. Descriptive Statistics 

Data analysis was accomplished using the Minitab 18.1 software (Minitab Inc., State College, PA, USA). The data were expressed as arithmetic mean ± standard deviation (SD). The figures were plotted as interval plots with standard error intervals. 

## 3. Results

### 3.1. Hybrid Coating Composition

**Figure 1:**
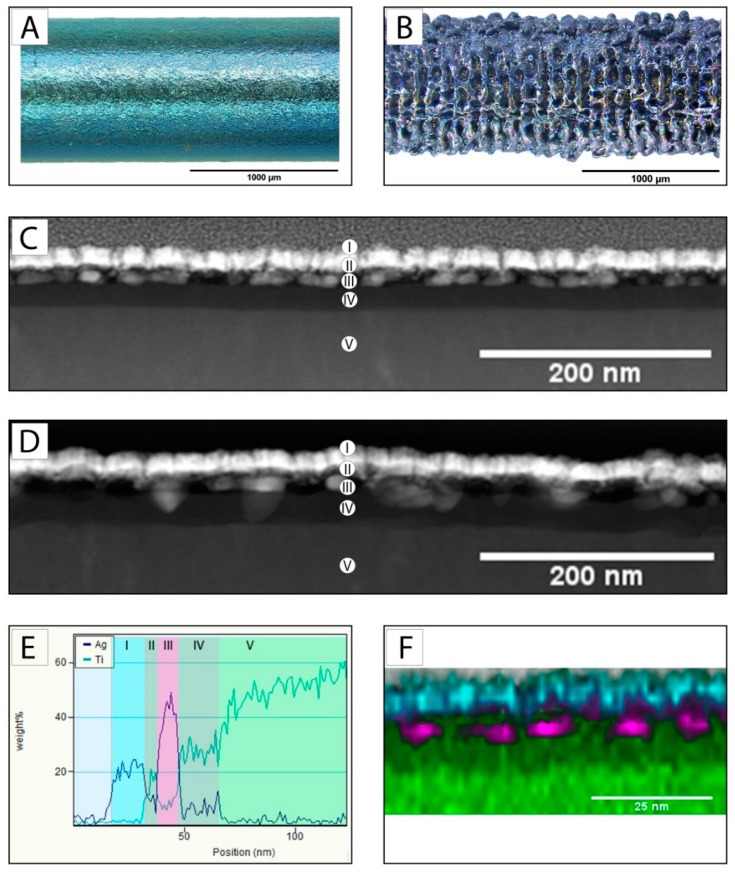
Optical micrograph of the U K-wire surface before (**A**) and after laser structuring (**B**); TEM of a cross-section (FIB) of the silver/titanium dioxide coating on titanium before (**C**) and after (**D**) 31 kGy gamma irradiation (coating composition: (I) surface marker, (II) titanium dioxide coating, (III) silver coating, (IV) titanium dioxide coating, and (V) titanium substrate); (**E**) energy-dispersive X-ray spectroscopy (EDX) line scan of a cross-section of the silver/titanium dioxide coating on titanium before gamma irradiation taken in vertical direction, and (**F**) EDX mapping after gamma irradiation: green represents titanium, purple is silver and cyan is platinum (surface marker).

The laser process increased the diameter from the U K-wire (Figure 1A) with a diameter of 0.99 mm (SD: 0.03) to 1.07 mm (SD: 0.02) for the PS K-wire (n = 15). The generated characteristic pore dimensions were 70–160 µm in length and 40–70 µm in width (Figure 1B). The size of the silver particles was approximately 10–30 nm with a diameter to length ratio of 1:2. The total coating thickness of the silver/titanium dioxide coating was 30–40 nm (Figure 1C,D,E). The coating composition showed no interference due to exposure to gamma irradiation with a dosage of 31 kGy. The silver particles were present as particles before and after the gamma irradiation (Figure 1C,D). EDX mapping confirmed this optical interpretation (Figure 1E,F). 

### 3.2. Gentamicin Release

**Figure 2:**
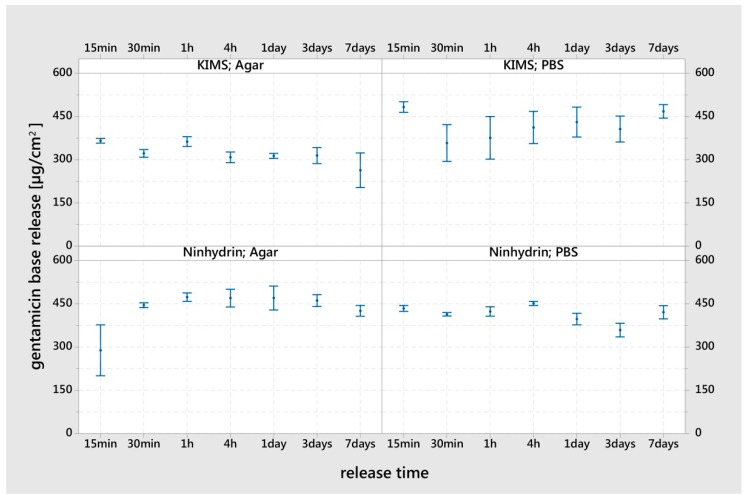
Gentamicin release *in vitro* over a period of seven days from the G hybrid K-wire pieces (loaded with 290 mg/mL gentamicin sulfate) incubated in phosphate buffered saline (PBS) or agar medium. The measurements were performed in individual replicates (ninhydrin n = 9, kinetic interaction of microparticles in solution (KIMS) n = 3). The results are displayed as mean values and standard errors.

Independent of the release system and measurement assay, the hybrid surface released the loaded gentamicin almost completely within 15 min. A delayed release of gentamicin was seen in agar medium using the ninhydrin assay for the 15 min time point only. The reference group (ninhydrin; PBS 4 h–7 days) showed a mean value of 430 µg/cm^2^ (SD: 33, n = 36) gentamicin base per square centimeter. An influence of PBS and agar in the KIMS assay was visible in the increased standard errors (PBS) and further reduced values (agar) of the interval bars (Figure 2). 

The gentamicin release capacity for the high concentration of the hybrid surface was 552.7 µg/cm^2^, SD: 19.9, n = 9. Autoclaving reduced this capacity to 394.1 µg/cm^2^, SD: 18.4, n = 9. 

### 3.3. Release of Silver 

The silver release was determined over 28 days in an agar medium (n = 3). The initial silver content was 3.7 µg/cm^2^ (SD: 0.1). After one day in the agar medium, the G hybrid K-wire showed a value of 3.2 µg/cm^2^ (SD: 0.3), decreasing to 3.1 µg/cm^2^ (SD: 0.4) at the seven-day time point and 3.1 µg/cm^2^ (SD: 0.1) after 28 days.

### 3.4. Antibacterial Efficacy (Silver)

**Figure 3:**
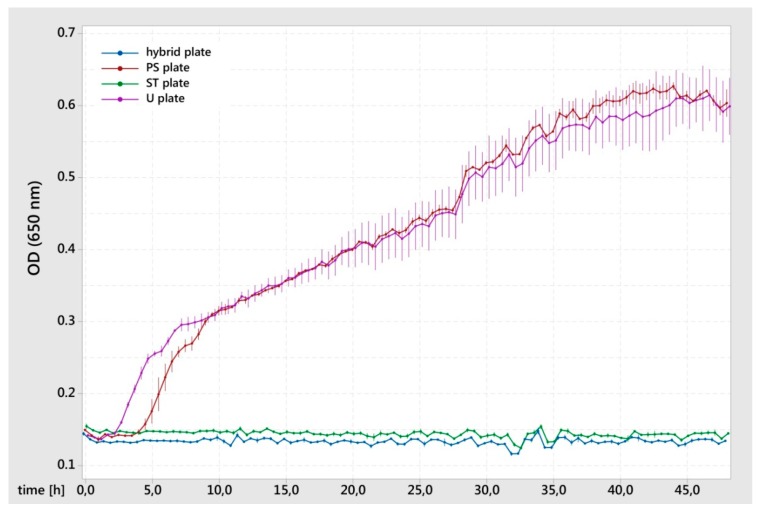
Proliferation of *Escherichia coli* after 24-h direct contact with the test surface. The means and standard errors of 3 (hybrid plates and ST plates) or 2 (PS plates and U plates) replicates are shown.

The *E. coli* growth curve showed no inhibition of proliferation for the U plates. For the PS plates, there were signs of a prolonged lag phase compared to the U K-wire. In comparison to these results, the hybrid plates and ST plates led to the complete inhibition of bacterial growth (Figure 3). Comparing the groups with and without silver, an antimicrobial efficacy of more than a 4 log reduction could be achieved. 

### 3.5. Antibacterial Efficacy (Hybrid Coating)

**Figure 4:**
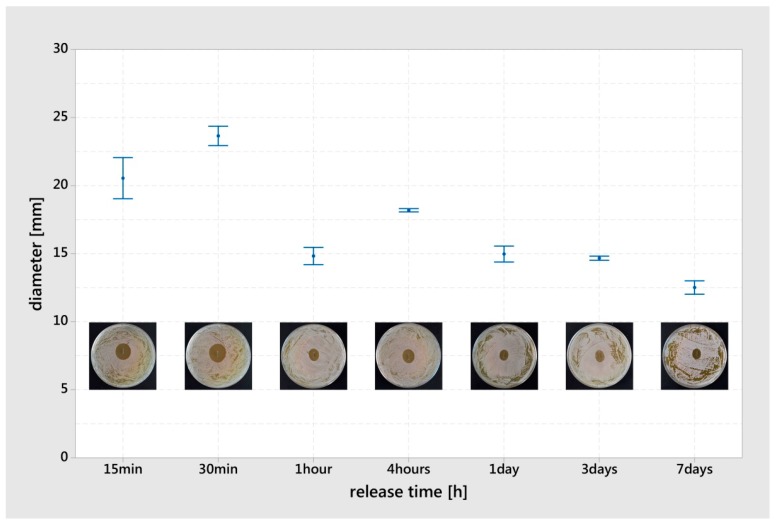
Zone of inhibition (ZOI) (*Staphylococcus cohnii*) generated from the G hybrid K-wire pieces after previous release in an agar medium. The results are displayed as mean values and standard errors (n = 3). A characteristic picture of the corresponding ZOI was integrated into the chart.

The generated ZOI of *S. cohnii* were caused by the gentamicin of the G hybrid coating. Prior to ZOI testing, the G hybrid K-wire pieces were used in gentamicin release experiments. At the early time point of 15 min, an inhibitory concentration of gentamicin was present to create a ZOI of 20.5 mm (SD: 2.1). The measured maximum ZOI was 23.7 mm (SD: 1.0) at a release time of 30 min. For the subsequent time points, the ZOI declined to an average value of 15.0 mm (SD: 1.9) (Figure 4).

### 3.6. Cytocompatibility 

**Figure 5:**
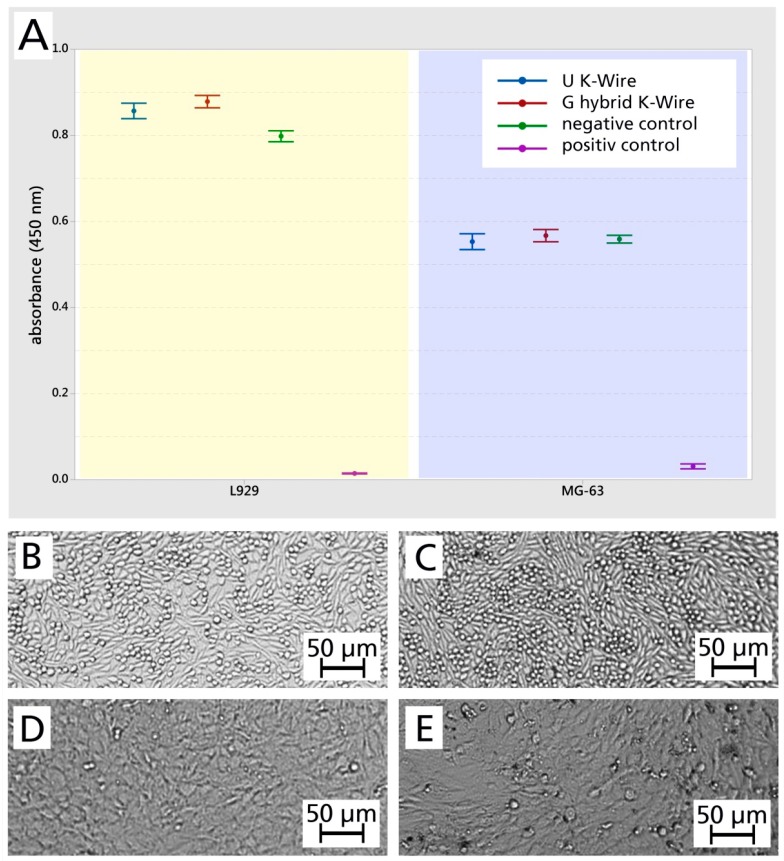
(**A**) Cytocompatibility testing (WST-1 assay). The results are displayed as mean values and standard errors (n = 3). (**B**–**E**) Optical micrographs of corresponding cells. L929 (mouse fibroblasts): negative control (**B**) and the cells that were in contact with the extraction medium of the G hybrid K-wire (**C**); MG-63 (human osteoblast-like cells): negative control (**D**) and the cells that were in contact with the extraction medium of the G hybrid K-wire (**E**).

The cell viability in the WST-1 assay of the G hybrid K-wire (loaded with 500 mg/mL gentamicin sulfate) was 103.4% for L929 (mouse fibroblasts) and 102.5% for MG-63 (human osteoblast-like cells) compared to the U K-wire (n = 5) (Figure 5A). The visual interpretation of optical micrographs (Figure 5B–E) were grade: 0 / reactivity: none. On the basis of these results, the loaded hybrid surface is to be considered as biocompatible according to the selected standard.

## 4. Discussion

The implant surface modification presented here combines two different local therapy approaches to dealing with implant-associated infections in one hybrid surface for medical implants. It was possible to develop a laser structuring process that allows a reproducible loading of antibiotics in an implant surface, which creates a reservoir for an initial local antibiotic burst release after implantation. The concept offers the possibility to load a sterile implant surface with an antibiotic or a combination, based on the results of an antibiogram of bacteria or for high-risk implantations, as preventive administration. 

Individually adapted patient therapy is an accepted procedure and clinical practice for bone cement [25]. Besides the antibiotic depot, the hydride surface can be complemented by metallic silver particles within a titanium dioxide coating. The titanium dioxide coating embeds the silver particles completely and hereby protects them from migration and mechanical stress. The antimicrobial mode of action for silver has been documented in several studies [26,27], as have the long-term release kinetics of silver ions [28], as shown in this study. Furthermore, Barres et al. [29] have discussed that a combination of silver ions and antibiotics will enable an increased antimicrobial activity compared to the separate use of the active substances, which additionally emphasizes the general concept of substance-combining hybrid coatings similar to the one shown here.

The porous structure of the hybrid surface was created via laser technology and acts as a gentamicin depot. Depending on the laser irradiation parameters, the laser’s interactions with the material (penetration depth, particle structure, topography) can be tailored. The selected laser parameter for this study generated a 40 µm thick porous structure, but smaller structures (150 nm thickness) can also be generated, as shown by Specht et al. [30]. 

In general, the quantity of the gentamicin in the hybrid surface used here could easily be adapted to higher (553 µg/cm^2^) or lower (430 µg/cm^2^) concentrations by adjusting the loading concentration. Those values are up to four to five times higher compared to the published results for, e.g., a polylactide coating with gentamicin (102 µg/cm^2^) [15] and are in a similar range as is used in palmitate-based coatings [31]. The reason for the reduced gentamicin base loading capacity of the hybrid coating after autoclaving (29% less) could be derived from the findings regarding surface wettability and an increased water contact angle due to autoclaving instead of gamma irradiation [32]. 

To receive a hybrid coating, the porous lasered structure was further modified using a PVD process to receive a silver/titanium dioxide coating. The dimensions of the sputtered silver particles (20–30 nm) were in accordance with the results for sputtered silver particles in conjunction with a siloxane matrix already described in the literature [33,34]. The silver particles remained in the same dimension and were not dislocated or ionized by the gamma irradiation (Figure 1). This is in contrast to the published results regarding the changes of silver particles due to gamma irradiation [35]. The stability observed in this study can be explained by the integration of silver particles within the titanium dioxide matrix and the exclusion of humidity and oxygen in the packaging during the gamma irradiation. The achieved silver concentration of 3.7 µg/cm^2^ was close to the concentration of the referenced anodization technique for therapeutic therapy (4 µg/cm^2^) [16] but was higher than the comparable prophylactic siloxane coating (1.5 µg/cm^2^) described by Khalilpour et al. [34]. 

Within the first day, the hybrid surface released 14% of the silver and by the subsequent time points (seven, and 28 days) had released 16% in total. The constant release values at time points seven days and 28 days can be explained by the fact that a chemical equilibrium was reached between metallic silver, silver oxide and silver ions in combination with the PBS solution present in the agar. According to measurements with comparable silver particles in a siloxane matrix, it is to be expected that the silver will be constantly released over time in an *in vivo* environment and will provide an antimicrobial effect for the local inhibition of bacterial colonization of the surface [34]. The silver/titanium dioxide coating (without gentamicin) showed a more than 99.99% reduction in the growth of *E. coli*, which is in the same magnitude as reported by Khalilpour et al. against *S. aureus* [34]. The prolonged lag phase for the PS K-wire could be due to differences in the surface topography that trigger bacterial adhesion and proliferation [36]. These results demonstrate the potential of the silver/titanium dioxide coating for the long-term prevention of bacterial colonization of the surface. 

The G hybrid K-wire released nearly all the loaded gentamicin within 15 min in the PBS. In addition, the release in the agar medium showed a minimally reduced release for the 15 minute time point. In general, it could be stated that the gentamicin release over time depends on the gentamicin formulation and storage technology. The release rate of gentamicin sodium dodecyl sulfate, gentamicin laurate and gentamicin palmitate is retarded, as found by Obermeier et al. [37]. This is compared to a direct release of gentamicin from a polylactide system whereby 50% was released within 10 min in PBS and 75% within 24 h [15]. The *in vivo* release of gentamicin in the polylactide system was measured as 70% within 24 h and 80% within 48 h after implantation [38]. Compared to the other systems, the hybrid surface described here showed the highest burst release in both quantity and time and no delayed minimal release at later time points, which would be preferable to prevent the development of antibiotic resistance due to the concentration being below the minimal effective concentration [39].

The examination of the ZOI over time (generated from the pre-released G hybrid K-wire) indicated an early availability of gentamicin due to the presence of 21 mm ZOI after 15 min release. Furthermore, the gentamicin content within the adhering agar after extraction and the remaining quantity in the hybrid K-wire increased at 30 min (ZOI 24 mm—Figure 4). Even if the *Staphylococcus* species cannot be directly compared, the antibacterial efficacy of the eluates from polylactide/gentamicin coatings was tested with *S. aureus* in a ZOI assay and showed bacterial inhibition over eight weeks [40]. Stobel et al. [40] also reported that the ZOI increase at the early time points (less than one day), reaching the maximum at one day at approx. 20 mm, and falling to 15 mm diameter. Beyond preventing bacterial colonization of the surface with silver, the early gentamicin release in high concentrations could have promising potential for the prevention of implant-associated infections at an early stage. 

The cytocompatibility of the hybrid surface could be demonstrated with different cell types such as fibroblasts and human osteoblast-like cells (Figure 5), even loaded with a 553 µg/cm^2^ gentamicin base and a silver concentration of 4 µg/cm^2^. This result is in line with the local application of gentamicin, which showed biocompatibility and no influence on bone healing *in vivo* [41], although high concentrations of gentamicin may impair bone regeneration [42]. Despite the cytotoxicity of a silver oxide hydroxyl apatide coating reported by Fielding et al. [43], the biocompatibility has been demonstrated *in vivo* for encapsulated silver particles [44]. Especially the immobilization of silver particles seems to be essential to generate a non-cytotoxic surface [45]. 

## 5. Conclusions

Implant-associated infections are a major concern in orthopedics. Even if systemic and local applications of antibiotics are available, their efficacy is very limited, particularly as implant surfaces cannot be customized according to the present infection. This hybrid surface design seems to be a promising approach in orthopedics to face these restrictions. Especially the possibility of individual administration of antibiotics in a comparable high dosage can be an advantage when fighting implant-associated infections in revision and trauma surgery. The addition of silver is a meaningful complement to provide a long-term protection of the implant surface during osseointegration. The achieved release behavior, efficacy and cytocompatibility met the objective of hybrid surface design. However, the limitation of *in vitro* testing makes further *in vivo* studies necessary to confirm the findings. Especially biocompatibility, osseointegration and efficacy have to be reviewed in *in vivo* environment before translation to applied clinical research.
